# Digital Dermatitis in Dairy Cows: A Review of Risk Factors and Potential Sources of Between-Animal Variation in Susceptibility

**DOI:** 10.3390/ani5030369

**Published:** 2015-07-13

**Authors:** Maeve A. Palmer, Niamh E. O’Connell

**Affiliations:** Institute for Global Food Security, School of Biological Sciences, Queen’s University Belfast, 18-30 Malone Road, Belfast, Northern Ireland, BT9 5BN, UK; E-Mail: niamh.oconnell@qub.ac.uk

**Keywords:** digital dermatitis, dairy cow, lameness, susceptibility

## Abstract

**Simple Summary:**

Dairy cow lameness is a major problem for the industry, causing reduced animal welfare and economic loss. Digital dermatitis (DD) is a bacterial disease causing painful lesions, generally on the heels of the rear feet, and is an important cause of lameness. There appears to be individual variation between animals in susceptibility to this disease. Particular physical, physiological and behavioural factors might influence individual susceptibility, but further work is required to clarify the influence of these factors and to determine how this information could be used to develop breeding and management strategies to reduce DD prevalence.

**Abstract:**

Digital dermatitis (DD) is a bacterial disease that primarily affects the skin on the heels of cattle. It is a major cause of lameness in dairy cows and a significant problem for the dairy industry in many countries, causing reduced animal welfare and economic loss. A wide range of infection levels has been found on infected farms, prompting investigations into both farm level and animal level risk factors for DD occurrence. There also appears to be individual variation between animals in susceptibility to the disease. The identification of factors affecting individual variation in susceptibility to DD might allow changes in breeding policies or herd management which could be used to reduce DD prevalence. Factors mentioned in the literature as possibly influencing individual variation in susceptibility to DD include physical factors such as hoof conformation and properties of the skin, physiological factors such as the efficacy of the immune response, and behavioural factors such as standing half in cubicles. Further work is required to determine the influence of these factors, identify the genetic basis of variation, clarify the level of heritability of DD susceptibility and to determine how this is correlated with production and health traits currently used in breeding programmes.

## 1. Introduction

Digital dermatitis (DD) is a bacterial disease that primarily affects the skin on the heels of cattle. Infection causes inflammation and skin damage, leading to pain and discomfort [1]. It is a major cause of lameness in dairy cows [2] and hence a significant problem for the dairy industry in many countries, causing reduced animal welfare and economic loss [3]. DD has recently been identified as an emerging issue in beef cattle in the UK [4] and the bacteria believed to cause DD have been identified in similar lesions in sheep (known as Contagious Ovine Digital Dermatitis; [5]), dairy goats [6,7] and even wild North American Elk [7]. In addition to this, these DD associated bacteria have been detected in three types of severe bovine foot lesions which have emerged during the last 15 years; toe necrosis, non-healing white line disease and non-healing sole ulcer [8]. These developments highlight the growing importance of DD for domestic and wild animals, and for farmers and veterinarians.

Despite the economic and welfare importance of this disease, many questions remain regarding its etiology, transmission, prevention and treatment. There are a number of reasons why the disease is proving difficult to deal with; firstly, the infection appears to be polymicrobial, with a variety of bacteria, particularly of the genus *Treponema*, isolated from lesions [9,10,11,12,13,14,15]. In addition, the bacteria involved initially proved difficult to grow in culture [13], experimental infection models have been difficult to develop [16,17], and the mechanisms of disease transmission have thus remained rather mysterious. Recent advances in laboratory methods have meant that good progress has been made in the identification of the most important pathogenic bacteria, and in the detection of these bacteria in the animals and in the environment of farms with DD infections [14,15,18,19].

A wide range of infection levels has been found on infected farms, prompting investigations into both farm/herd level risk factors and animal level risk factors (for example parity and stage of lactation) for DD occurrence (e.g., [20,21] summarised by [22]). Both the farm level and animal level risk factors can provide useful information when trying to minimise DD infection levels and understand when risks of infection are highest. An interesting, but less investigated, aspect of DD is that there appears to be individual variation between animals in susceptibility to the disease; Laven [23,24], Capion *et al.* [24] and Gomez *et al.* [25] all found that some animals within a herd were infected repeatedly while others of the same breed and parity and kept under the same conditions were never infected. If the reasons for this individual variation in susceptibility could be identified, they could add to our understanding of the disease and contribute to the search for effective prevention and treatment methods.

This review will begin by providing an overview of the disease and the problems that it causes, focusing where possible on new developments reported in the literature. It will then briefly outline the farm-level and animal-level risk factors for DD that have been described in the literature. Finally, it will introduce research that has been carried out into individual variation in susceptibility to DD.

## 2. Background

DD was first described in Italy in 1974 [26] but has since been recorded in the majority of countries with a dairy industry [27]. Infection causes ulcerative lesions along the coronary band [26] or on the skin adjacent to the interdigital cleft [28], leading to pain and discomfort [1]. The course of the disease is generally described as according to Dӧpfer *et al.* [29], who split it into four stages. The early lesion or M1 stage is a circumscribed granulomatous area that is normally small in size [29] and is not generally painful [28]. The lesion then develops into the M2 or classic ulcerative stage [29]. This stage of the lesion is generally larger and is painful on palpation [28]. Once the lesion begins to heal (which is not normally until after treatment), a scab forms over the ulcerated area and the lesion is described as an M3 [29]. In some cases the lesion progresses to an M4 or chronic stage [29] which is characterised by surface proliferation or dyskeratosis. This stage is generally not painful but is infectious and can transition back to an active M1 lesion [30,31]. The M4.1 lesion type was added by Berry *et al.* [31] to describe a chronic lesion (M4) with a small, painful area of active M1 lesion within it. Once a lesion has completely healed and only healthy skin remains it is classified as an M5 [31]. It is common to find animals with lesions on both rear feet concurrently; Laven [23] found that 51% of animals in one herd had lesions on both feet, 22% on the left foot only and 27% on the right foot only. It is not known why some animals develop DD on only one hind foot when both feet are exposed to the same risk factors, and investigation of the factors influencing which foot becomes infected (or which is infected first) could contribute to the search for factors which affect variation in susceptibility to the disease.

The presentation of DD varies geographically, with a proliferative form more common in the USA and a more erosive form predominant in Europe [32]. The disease is generally referred to as papillomatous DD (PDD) in the USA and digital dermatitis (DD) in Europe [32], however during this review the term digital dermatitis (DD) will be used to refer to both conditions.

### 2.1. Etiology and Transmission

Although a lot of questions still remain, recent advances in laboratory techniques such as gene sequencing have begun to provide the answers to essential questions regarding the identification of causative bacteria and the transmission of the disease. Spirochaete bacteria of the genus *Treponema* are consistently found in DD lesions (e.g., [9,10,12,14,15,33,34]) and are thought likely to be the primary aetiological agents of DD [35]. Their presence deep within lesions [36] suggests that they are invasive and not simply colonising damaged tissue [37]. Treponemes have been isolated from tissue-destructive disease lesions from a number of different hosts, including humans [37]. Treponemal diseases in humans include venereal and endemic syphilis, bejel, yaws and pinta, which all have a relapsing clinical course and include (but most are not restricted to) skin lesions [38]. Treponemes have also been implicated in human periodontitis, which is also thought to have a polymicrobial etiology [37], and indeed some of the treponemes identified from DD lesions show strong similarities to those which are involved in periodontitis [10]. Although treponemes are the most commonly found bacteria in lesions, other types of bacteria have also been identified including *Borrelia burgdorferi* [36,39], *Bacteroides* and *Mycoplasma* species [39], *Campylobacter* species [29] and *Candidatus Amoebophilus asiaticus* [15].

Recently, work has been carried out to characterise the microbiome of healthy skin and DD lesions at different stages of infection [14,15]. Krull *et al.* [14] examined the microbiota of lesions at seven different stages of development and found that there was a dramatic increase in the proportion of treponemes detected in lesion biopsies as the lesions developed. They also found that the proportions of the different treponeme species identified changed as the lesions developed. This agrees with the findings of Zinicola *et al.* [15], who found that the microbiome of skin with active DD lesions (M1, M2 and M4.1) was distinct from the microbiome of skin with inactive lesions (M3 and M4) and from healthy skin. The microbiome of active, ulcerative DD lesions was dominated by six groups of treponemes; *Treponema denticola*, *Treponema maltophilum*, *Treponema medium*, *Treponema putidum*, *Treponema phagedenis* and *Treponema paraluiscuniculi* [15]. The findings of these two studies suggest that some of the variation in bacterial species found in DD lesions between previous studies could be due to samples being taken at different stages of lesion development.

The advances in laboratory techniques which are helping to clarify the causative agents of DD are also helping to show how the disease might spread between animals. Although environmental transmission of DD-causing treponemes has been postulated for many years, these bacteria initially proved hard to find in the environment, with Evans *et al.* [18] not finding any evidence of them in dairy cow faeces or environmental slurry samples. Since then, advances in technology have allowed the detection of treponemes in both dairy cow faeces and environmental slurry samples on infected farms by using a targeted deep-sequencing approach to detect very small amounts of bacterial RNA [19]. The role of the gut as a possible reservoir of DD bacteria was postulated by Shibahara *et al.* [40], who found that similar spirochaetes appeared to be responsible for simultaneous infections of bovine dysentery and DD in two Japanese dairy cows. Since then, Evans *et al.* [18] detected DD-related treponemes in both oral and rectal tissues of dairy cows on DD-affected farms, and these findings have been confirmed by Zinicola *et al.* [15], who found DD-associated treponemes in both the rumen and faecal microbiomes. These findings suggest that the gastrointestinal tract acts as a reservoir for DD-related treponemes [15] and faeces/slurry as a mode of transmission between the reservoir and site of infection [18]. Direct skin to skin transmission from infected to uninfected feet has also been suggested as a possible route of infection [18], as has transmission via hoof trimming implements [41].

### 2.2. Treatment and Prospects for Vaccine Development

Treatments for DD include systemic and topical antibiotics [1,9] and are summarised by [22]. In herds where a high proportion of animals are infected with DD individual treatment is very time-consuming, so many farmers instead use footbaths to treat the entire herd [1]. Unfortunately, elimination of DD is rarely seen, so repeated application of treatments is required to prevent recurrence of infection [2]. An antibody response is produced by dairy cows when they are infected with DD [33,42], however this response does not seem sufficient to prevent further infections, as some animals are infected repeatedly [42]. One reason suggested for the difficulties encountered when trying to treat DD and prevent recurrence is the fact that a number of the treponemes thought to be involved in DD have been shown to have encysted as well as spiral forms [43]. It is possible that these encysted forms of the bacteria could persist deep within the lesions and cause a recurrence of clinical disease at a later date, though more research is required to determine the significance of the encysted form of the bacteria and its response to DD treatments [43].

There have been a number of efforts over at least 15 years to develop a vaccine to DD, however none are currently available. Two studies have found promising results. In the first, Keil *et al.* [44] found that adult cows and heifers vaccinated with an inactivated *Treponema* bacterin showed a significant reduction in PDD prevalence compared to unvaccinated control animals. In the second, Berry *et al.* [45] report a study in Nebraska where a different *Treponema* bacterin marketed as TrepShield (Novartis Animal Health) led to a significantly lower occurrence of DD in vaccinated animals when heifers were immunised before calving and cows immunised during the dry period, however this vaccine has since been withdrawn.

The results of other trials have been less encouraging. Berry *et al.* [46,47] vaccinated and then experimentally infected calves with four species of bacteria and found mixed results—the diameter of lesions was reduced in vaccinated calves in one experiment but not in another and no antibody response to the vaccine developed in either experiment. Fiddler *et al.* [47] report the findings of a study which evaluated a vaccine containing *Serpens* sp. bacterin, developed by a commercial company. They found that, although the dairy cows involved in the trial did develop an immune response to the bacterin, there was no reduction in the prevalence or severity of DD infections among vaccinated cows when compared to controls. They therefore concluded that the vaccine did not show any clinical efficacy in terms of DD prevention [47]. Work is ongoing to develop a vaccine which will be effective in the prevention of DD. For example, Staton *et al.* [48] are currently using a reverse vaccinology approach to identify vaccine candidate proteins for a recombinant vaccine which they hope will be effective against treponemes from three different phylogenetic groups.

### 2.3. Consequences of DD

The majority of active DD lesions are painful [1,21,49], so the primary consequence of DD infection is likely to be pain. If the DD is not treated, infected animals can remain lame for up to four months [50], implying ongoing pain and discomfort. Animals that are in sufficient pain to develop lameness also show other changes in behaviour when compared to sound animals, such as an increase in lying time [51,52,53,54] and shorter total feeding time [51,53,55,56], which in turn might be expected to have some effect on productivity.

Studies that have examined the effect of DD on milk yield have given differing results and, although it is a major cause of lameness, DD infection does not always appear to be associated with a significant reduction in milk yield. Amory *et al.* [57] found that animals with DD in England and Wales did not show a decreased milk yield during infection, but had a slightly increased milk yield after treatment. In two other studies, animals with DD produced less milk, but the difference was not significant [58,59]. Warnick *et al.* [60] found that animals with DD on two US dairy farms showed a reduction in milk yield, but found that this reduction was not as large as for animals with some other causes of lameness. Using a large data set from Holstein cows on French farms, Relun *et al.* [61] found that DD caused a small but significant decrease in milk yield (<1 kg per day). Pavlenko *et al.* [62] found that DD-affected Swedish Red and Swedish Holstein cows had a significantly lower milk yield (5.5 kg energy corrected milk per day) than healthy control cows. Gomez *et al.* [25] found that the DD infection history of heifers affected their milk yield during the first lactation; a reduction in milk yield of 199 kg or 335 kg milk over 305 days was found for cows that had experienced one or more than one DD infection before their first calving respectively, when compared to cows that had not experienced a DD infection before calving.

Although there is evidence that lameness in general impairs fertility (e.g., [63,64,65]), there has been very little work relating DD infection specifically to changes in fertility. An increase in the calving to conception interval and the number of days open was seen in Mexican Holstein-Friesian cows with DD compared to healthy cows [58]. In agreement with this, Gomez *et al.* [25] found that heifers repeatedly infected by DD before their first calving had a lower conception rate at first service and an increase in the number of days open when compared to cows that had not had any DD infection before first calving. Overall, Zinicola *et al.* [15] estimate that DD leads to an annual economic loss of US $1.1billion from dairy cows in the US and EU if a DD prevalence rate of 25% is assumed.

## 3. Farm Level Factors Affecting Susceptibility to DD

A number of studies conducted across different countries have examined farm level risk factors for DD (e.g., [21,66,67,68,69]). The main risk factors found relating to housing and general management are briefly discussed below.

### 3.1. Housing

The majority of the research that has examined the relationship between pasture or confinement and DD has found that increased access to pasture is associated with a decreased risk of DD [21,32,68,70]. In contrast to this, one study found that access to pasture was associated with an increased risk of DD [69]. However the authors suggest that this relationship may in fact be an effect of DD; caused by herdsmen providing additional access to pasture when it is considered that the level of claw disorders is high.

The type of housing provided for dairy cows has also been linked to DD prevalence. Laven [23] showed that cattle housed in straw yards had less, and less severe, DD than those housed in cubicles. Onyiro *et al.* [70] also found that cubicle housed animals were more likely to suffer from DD than animals in straw yards or at pasture. Within cubicle houses, longer and wider cubicles were found to reduce the risk of the disease [21]. A suggested connection between cubicle size and risk is that if cubicles are too small, animals are likely to spend more time standing, which increases the contact between their heels and slurry [71].

A number of authors have found links between the cleanliness of the environment and DD prevalence. In fact, Relun *et al.* [72] found a direct relationship between levels of leg cleanliness in dairy cows (measured at the herd level) and levels of DD; herds in which animals had dirtier legs had a higher prevalence of DD. Solid floors composed of grooved concrete were identified as a risk in the UK [73] and in the USA [68] when compared to textured concrete floors. This could be due to the fact that a small amount of slurry remains in the grooves of this type of flooring even when it is freshly scraped [73]. A common theme throughout the housing factors that have been linked to DD is the level of contact between the cows’ feet and slurry, which has been highlighted as an important risk factor for DD [23]. In addition, the moisture level in the environment (which could be affected by both the efficiency of slurry removal and building ventilation) could also be important as DD has been associated with damp conditions both in cubicle houses [20] and in corrals [32].

### 3.2. Biosecurity—External Hoof Trimmers and Buying in Replacements

The introduction of new animals to the herd, primarily through buying in replacement heifers, has been found to be associated with DD prevalence by studies in the USA and Chile [66,67,68]. Certain hoof trimming practices have also been highlighted as a biosecurity concern and have been linked to DD prevalence. These include the use of professional hoof trimmers (who work on a number of different farms), and hoof trimmers who do not clean the hoof trimming equipment between animals [68]. Evidence in support of the theory that the bacteria causing DD could be spread by hoof trimming equipment was recently provided by Sullivan *et al.* [41], who found that treponemes from at least one of the three phylogroups associated with DD (*T. medium*/*T. vincentii*-like, *T. phagedenis*-like and *T. denticola*/*T. putidum*-like) were present in at least 42% of swabs from knives used to trim the hooves of cattle infected with DD. Disinfection of the knife with an iodine disinfectant greatly reduced the proportion of swabs that tested positive for the treponemes, but did not eliminate the treponemes entirely (with 24% of swabs from disinfected knives testing positive for at least one phylogroup). A number of papers have, however, found that hoof trimming was associated with a reduced risk of DD occurrence [21,69,72]. Relun *et al.* [72] suggest that this could be because the DD lesions were treated at hoof trimming visits and then recovered quickly, or because hoof trimming could have improved the conformation of the feet, making the animals less susceptible to DD (as discussed in Section 5.3).

### 3.3. Diet and Nutrition

Feeding strategy and nutritional content of the diet have both been identified as possible risk factors for DD. The rate at which concentrate supplementation is increased for dairy cattle after parturition has been linked to DD prevalence; Somers *et al.* [21] found a higher incidence of DD in herds where the maximum level of supplementation was reached at two weeks post-partum (compared to at three weeks). The authors suggested that this could be due to an increased metabolic imbalance leading to a greater susceptibility to disease.

Gomez *et al.* [74] recently investigated whether the beneficial effects of dietary supplements on immune responses and skin quality might reduce the incidence of DD. They supplemented Holstein steers with higher than standard levels of organic trace minerals and iodine and then monitored their responses to natural DD infection and experimental infection. No natural DD infections occurred during the trial, and the infection rates following experimental infection were lower than predicted. Therefore, no significant differences were found between the control and treatment groups, however there was a trend for the supplemented animals to have a reduced DD infection rate and also a trend for the average size of the experimentally induced lesions to be smaller in this group [74].

### 3.4. Management Practices

A number of general farm characteristics and management practices have been associated with the risk of DD. Two studies have found that the risk of DD increases with increasing herd size [66,68]. The way in which animals are grouped has also been found to have an effect; Somers *et al.* [21] found that the incidence of DD was reduced if heifers were housed with the lactating dairy cows for a prolonged time before calving, which the authors suggested gave the heifers more time to adapt to the environment. The same study also found that housing dry cows with the milking herd before calving actually increased their risk of DD; risk was lowest if they were introduced to the milking herd immediately after calving [21].

## 4. Animal Level Factors Affecting Susceptibility to DD

A number of risk factors have also been reported which identify groups of animals within a herd that are at a higher risk for DD than others; animals of particular parity, animals at particular stages of lactation and animals of a particular breed. These factors are likely to affect all animals within the group in a similar way, however there is some variation in the literature in terms of which parity of animals and stage of lactation are associated with highest risk of DD infection, as discussed below.

### 4.1. Parity

A number of studies have found that first parity animals are most at risk of developing DD [21,32,67]. It has been suggested that the increased prevalence in this group is caused by the stress related to the significant environmental and metabolic changes that heifers experience around the time of their first calving [21,32,67]. In contrast to this, two studies have found that second lactation animals are the most likely to suffer from DD [58,69]. There may be management differences affecting which parity is most at risk. For example some farms may keep heifers separate from the main dairy herd for the first lactation, meaning that they do not experience the stress of mixing with the older cows until the beginning of the second lactation. Somers *et al.* [21] and Holzhauer *et al.* [69] both found that the risk of DD declined with each parity after the second lactation. The fact that there is such a high recurrence rate of DD lesions suggests that the lower incidence in later lactations is not due to the development of immunity to the disease [32]. A contributory factor to the decline in lesion prevalence with age may be that older animals with lesions are culled [67]. If DD susceptibility is linked to hoof conformation (see Section 5.3) then there may be a further reason for prevalence declining with parity as heel height increases as animals age [75].

### 4.2. Stage of Lactation

It is generally agreed that cows in the dry period have a lower risk of developing DD than lactating cows [21,28,58,69]. Dry cows are generally fed a different diet which may lead to more solid faeces, reducing the contact between the cows’ heels and liquid slurry [21]. There is not yet a clear answer, however, to the question of which period during lactation animals are most susceptible to DD. Argaez-Rodriguez *et al.* [58] found that the highest risk for new infections was in the first month after calving. There is immune suppression in the peri-partum period making animals more susceptible to disease [76], and this may be enhanced in cows experiencing metabolic stress [77]. These factors could, in turn, contribute to higher levels of DD [21]. However, Holzhauer *et al.* [69] found that animals were most at risk during peak lactation. The high energy diet fed at peak lactation has been suggested as a contributing factor to the high prevalence of DD lesions at this time—a high energy diet can lead to larger quantities of a more liquid faeces being produced and hence more contamination of the heels with slurry [21,69,71].

### 4.3. Breed

A number of studies have found differences between breeds in lameness and DD prevalence, and many of these have found that Holstein-Friesians (and their cross breeds) are more susceptible than other breeds. For example, Barker *et al.* [73] found that farms with no Holstein-Friesian cows had a lower prevalence of lameness than farms with only Holstein-Friesians. In agreement with this, Holzhauer *et al.* [69] found that purebred and crossbred Holstein-Friesian cows had a higher risk of DD than a dual purpose breed (MRIJ), Rodriguez-Lainz *et al.* [67] found that Holstein x German Black-Pied animals had a higher prevalence of PDD than German Red-Pied animals in Chilean dairies, and Relun *et al.* [72] found that Holsteins were more likely to develop DD than Normande or other breeds on a group of French dairy farms.

## 5. Individual Factors Affecting Susceptibility to DD

Individual differences in susceptibility to a number of infectious diseases have been investigated in dairy cows; for example mastitis [78,79], uterine diseases [80] and Johne’s disease [81,82]. Variation in susceptibility to mastitis has been particularly well researched, and a number of factors which influence individual susceptibility have been identified [78]. These factors are both morphological, for example udder shape and placement [83], and physiological, such as aspects of immune function (e.g., [84,79]. It could be expected that individual variation in susceptibility to DD would also be affected by morphological and physiological factors. As it seems likely that the transmission of DD involves reservoirs of infection in the environment [15,19] and might also occur by direct cow-to-cow transmission [18], it seems pertinent to also consider the behaviour of the animals as a source of individual variation in susceptibility, as this could affect their exposure to environmental reservoirs and infected animals.

The majority of morphological, physiological and even behavioural factors which could influence individual susceptibility to DD are likely to be influenced to a greater or lesser extent by genetics. This means that there are two possible strategies which can be used when trying to identify individual differences which affect susceptibility; searching for the underlying genetic differences or examining the morphological, physiological or behavioural changes that these genetic differences produce or contribute to (phenotypic differences). The majority of work carried out to date into DD susceptibility has focused on phenotypic differences, however the rapid advancement of genomics makes it a promising area for future research, allowing precise determination of where genetic differences lie. This section will begin by outlining research that has been carried out into the heritability of DD and the use of genetic approaches in the investigation of individual differences in susceptibility. It will then go on to outline the aspects of variation in some physical (hoof conformation, properties of the skin), physiological (immune response) and behavioural characteristics which could contribute to individual differences in susceptibility to DD.

### 5.1. Heritability of DD

Genetic heritability values found for DD in Holstein-Friesian dairy cows differ quite widely between studies. van der Waaij *et al.* [85] calculated a heritability of 0.10 for DD in Dutch Holstein-Friesian dairy cows, considerably higher than the value of 0.029 calculated by Onyiro *et al.* [70] for DD in British Holstein-Friesian dairy cows. It is suggested by Onyiro *et al.* [70] that this difference could be due to the difference in models used to estimate heritability or to the size of the dataset used (over 90,000 records used by Onyiro *et al.* [70] compared to approximately 22,000 used by van der Waaij *et al.* [85]). A substantially higher heritability of between 0.33 and 0.40 for DD was found by Oberbauer *et al.* [86], who used repeated observations on 5043 Holstein-Friesian cows over a number of lactations on three dairy farms in California, USA. The authors suggest that the similar production systems used on the three farms (freestall housing) and the consistent classification of lesions by trained foot trimmers could have contributed to the higher heritability that they found [86].

Although there is variation between studies in the estimated heritability of DD, the fact that all of the studies have found a heritable component demonstrates that there is scope to selectively breed for a reduction in DD susceptibility. The consequences of selection for a reduction in DD are, however, not clear as these is no agreement as to the genetic correlations between DD and milk yield. Onyiro *et al.* [70] found that presence of DD was associated with lower lifespan and decreased milk and fat yield, so suggested that breeding for reduced DD could be accompanied by an improvement in these traits. More recently, however, Oikonomou *et al.* [87] found that cows whose sires had high genetic merit for milk and protein production were at higher risk of developing DD.

### 5.2. Genetic Approaches to Investigating DD Susceptibility

Scholey *et al.* [88] investigated variation in individual susceptibility to DD from a genetic perspective, by looking for single nucleotide polymorphisms (SNPs) which differed between animals that had a history of DD and those that appeared unaffected by DD. The study found eight SNPs that were associated with DD disease status, some of which were located in genes which could have a plausible role in DD pathology, such as skin cell proliferation and inflammatory responses [88]. It has been suggested that this type of study could be repeated with larger sample sizes as the costs of genotyping decrease [88] and that the information gained from such work could be used in screening programmes as part of targeted breeding strategies to reduce the number of animals susceptible to DD [89].

### 5.3. Hoof Conformation

One of the first physical attributes investigated with respect to variation in DD susceptibility was the conformation of cows’ hooves. There is considerable variation in hoof conformation within breeds of dairy cattle and at least a proportion of this variation is heritable [90]. As hoof conformation could affect the amount of contact between heels and slurry or damp underfoot conditions (shown to be important for the establishment of DD infection by [16,17], a number of studies have been carried out to examine relationships between hoof shape and DD occurrence. Although a pilot study by Walker *et al.* [91] did not find any relationship between DD presence and claw length, claw width or claw angle (the slope of the dorsal border of the claw with respect to the floor surface [75]), a number of studies since then have found relationships between aspects of hoof conformation and DD.

Laven [92] carried out a preliminary study of DD and hoof conformation in 20 Holstein heifers, monitoring their DD status from three months of age until 12 weeks after calving and making hoof conformation measurements 24 weeks after calving. He found that at this stage the mean heel height was lower and mean toe length was longer for animals that had DD than for animals that did not [92]. In agreement with this, lower heel height associated with a higher prevalence of DD was also found by Olechnowicz and Jaśkowski [93] in a study of 144 primiparous Polish Holstein-Friesian cows. Laven [92] suggested that lower heels could lead to a higher risk of DD by increasing the amount of contact between the skin on the heels and slurry. Not all findings of the two studies agreed, however, as Olechnowicz and Jaśkowski [93] also found that a higher risk of DD was associated with a shorter dorsal length (distance between the dorsal border and the tip of the toe).

Baird *et al.* [94] examined a total of 84 Holstein-Friesian and Norwegian dairy cows in three production systems (grass based; housed and fed a low concentrate diet; housed and fed a high concentrate diet) in Northern Ireland. They found that animals with DD had wider heels than animals without DD, but did not find any relationship between heel height or claw length and DD occurrence. There has also been some interest in the role that could be played by the interdigital cleft; Daniel [95] found a pattern of increasing DD prevalence with decreasing width of the interdigital cleft in Holstein cows and postulated a link between DD development and the more anaerobic conditions that a narrow interdigital space would produce.

For the purpose of this review, one drawback of the studies discussed above [92,93,94,95] is their cross-sectional design, which means that it was not possible for them to demonstrate that any differences observed were likely to be the cause (rather than an effect) of DD [92,96]. A longitudinal study of the effect of DD on hoof conformation has recently been carried out by Gomez *et al.* [96] using a cohort of 644 Holstein heifers. Monitoring the animals for DD lesions and taking three sets of hoof measurements over approximately six months (starting at a mean age of 540d), they found that M2 (active) DD lesions were associated with increased heel height, claw angle and depth of the interdigital cleft (the distance between the skin-horn junction of the lateral and medial heels and the proximal end of the skin fold at the plantar aspect of the foot [96]). The increased heel height found in this study is attributed to the fact that cows with DD tend to take weight off their heels and onto their claws, which increases wear of the toes and reduces that of the heel, thus increasing heel height over time [92,96]. The same process is likely to result in the increased claw angle observed [96]. A greater claw angle was found in animals with DD than in animals without DD by Olechnowicz and Jaśkowski [93] and the findings of the Gomez *et al.* [96] study suggest that this could be due to DD rather than a predisposing factor. The increase in the depth of the interdigital cleft associated with active DD lesions was thought by Gomez *et al.* [96] to be caused by inflammation and the prominence of the DD lesion. These changes caused by the active lesion could cause a more anaerobic environment and potentially facilitate further bacterial growth [96].

It is clear that studies investigating the relationships between hoof conformation and susceptibility to DD have not found consistent results and there are a number of factors which could contribute to this, including the small sample sizes used in many of the studies, the varied housing and management systems and the complicating point that DD has now been shown to alter hoof conformation as lesions progress [96]. Further, larger-scale research in this area could help to clarify whether some aspects of hoof conformation, including heel height and the morphology of the interdigital cleft, are associated with individual susceptibility and whether relationships are similar in different breeds and housing systems.

### 5.4. Properties of the Skin and Hair Follicles

A second physical characteristic which has been proposed as a possible cause of differences in susceptibility to DD is the efficacy of the skin in preventing pathogen entry [97]. How well the skin acts as a barrier to particles and pathogens can vary between individuals and is affected by a number of factors including the thickness [98] and lipid content [99] of the stratum corneum layer (the top layer of the epidermis). The barrier function can also be affected by single gene mutations with large effects; for example some mutations in the gene that codes for the protein filaggrin (important in correct stratum corneum formation) have been shown to reduce barrier function and are associated with the occurrence of atopic dermatitis in humans [100].

In a small study comparing the skin permeability of dairy cows with and without a history of DD, Palmer *et al.* [97] did not find any difference in the permeability of skin to a dye solution (methylene blue). The study also tested the effect of prolonged (24 hour) contact with slurry on the permeability of the skin and found that slurry increased skin permeability to the dye, but that there was no difference in the magnitude of the increase in permeability between animals with and without a history of DD [97]. These results imply that in the population investigated, differences in susceptibility to DD were unlikely to be caused by simple differences in the skin barrier function (as is the case in some humans with atopic dermatitis and accompanying skin infections [101]).

There has been growing interest in recent years in the role that the hair follicles might play in the movement of particles across the skin barrier [102,103,104]. The stratum corneum is not entirely continuous around the base of hair follicles which means that there are areas where it is easier for particles to pass through into the lower layers of the skin [104]. In relation to DD, it has been suggested that treponemes might use the hair follicles as a means of entry to, or exit from, bovine skin, as electron micrographs of DD-infected tissues have shown large numbers of treponemes in the hair follicles and radiating out towards deeper parts of the tissues [34]. The possible role of hair follicles in the development of DD lesions has been further highlighted in a study by Scholey *et al.* [105] which examined changes in gene expression within host tissues during DD infection. They found that there was altered expression of a number of genes relating to epidermal barrier formation within DD lesions and they suggested that this could reduce the efficacy of the barrier between the hair shafts and skin [105].

Hair follicles can also act as a reservoir for bacteria and therefore a source for re-colonisation after treatment and disinfection [106]. If this is the case for DD it would have implications for treatment, as it would highlight the importance of getting any treatment solutions deep into the hair follicles. It might also be possible to develop treatments aimed specifically at the hair follicles which could then act as reservoirs of antibacterial activity, as has been investigated for *Candida albicans* infection in human skin [107]. Considerable variation has been found between individuals in the number of hairs per unit area in Brazilian Holstein cattle (a range of 221 to 3545 hairs per cm^2^, [108]). A proportion of the variation in number of hairs per unit area does appear to be heritable in cattle [109]. Therefore, if a strong relationship was found between the number of hair follicles per unit area and susceptibility to DD, it might be possible to select for animals that had more or less hair follicles, as appropriate.

### 5.5. Immune Response

The immune response seems an obvious candidate to examine when looking for sources of variation in susceptibility to a bacterial disease, for example variation in susceptibility to mastitis has been linked to variation in a number of different aspects of the immune response (summarised by [78]). Unfortunately, it seems that dairy cows do not produce an effective immune response to DD: once a cow has had DD, it is significantly more likely to get it again and previous exposure of heifers to an environment where DD is endemic does not appear to reduce their risk of developing the disease after their first calving [71]. Although there has not been much work to date linking susceptibility to DD with variation in the immune response, work is ongoing to develop a better understanding of the immune response to DD, which may point the way to aspects that could influence variation in susceptibility.

There is clear evidence that animals do produce antibodies against treponemes that are associated with DD infection. For example, Walker *et al.* [110] found that levels of antibodies to two spirochaetes associated with PDD were significantly higher in animals with PDD lesions than in animals with no PDD lesions on a California dairy farm. Vink *et al.* [111] developed an ELISA using five Treponema antigens, including representatives from each of the three main serogroups identified in a survey of DD lesions in the UK, and they also found that lesion positive animals had higher antibody titres than lesion negative animals. Recently, Gomez *et al.* [42] developed an ELISA to detect anti-*Treponema* IgG antibodies and found that blood titres of this antibody in heifers increased by an average of 56% after a diagnosis of a first clinical case of DD. The antibody levels decreased to baseline levels within six months for heifers who were treated and did not have a recurrence of the disease, but in heifers with recurring lesions the antibody levels remained high [42].

Although antibodies are produced, the acquired immune response does not seem effective at preventing re-infection with DD [71]. Therefore it may be that if there are any immune differences in susceptibility, they are more likely to be linked to the innate immune response. A poorer innate immune response has been associated with increased susceptibility to infectious diseases in general [76]. Indeed, a number of the SNPs found by Scholey *et al.* [88] to differ between susceptible and non-susceptible animals were found to be in genes related to the inflammatory response, part of the innate immune system.

Another area where individual differences could affect DD susceptibility is in the extent to which the host immune responses are modulated by DD associated treponemes. Recent research has begun to characterise the interactions between treponemes and host tissues within DD lesions. Scholey *et al.* [105] used RNA-Seq technology to compare gene expression in skin biopsies from DD lesions and healthy tissue in Holstein-Friesian dairy cows. It was found that within the lesions there was altered expression of a number of genes relating to immune function and this led the authors to hypothesise that these changes could facilitate the development of DD by reducing the efficacy of the immune response [105]. Refaai *et al.* [112] also examined samples from various DD lesion stages and from healthy skin. They found that the transcription of Interleukin-8 (IL8), a chemokine, was upregulated in the keratinocytes of samples from M2 and M3 lesion stages. IL8 is involved in the inflammatory response and also in keratinocyte proliferation, as seen in the skin condition psoriasis [113], hence it is suggested that its upregulation could help to explain the hyperplasia and thickening of the epidermis observed in DD lesions [112]. As variants of IL8 receptors and promoters have been identified and associated with susceptibility to other infectious diseases, Refaai *et al.* [112] suggest that the role of IL8 in the pathogenesis of DD should be further investigated.

### 5.6. Behaviour

It is possible that differences in the behaviour of dairy cows could affect their susceptibility to DD. Differences in dairy cow behaviour have already been linked to susceptibility to non-infectious conditions such as claw horn lesions [114,115]. However, as DD is an infectious disease, the ways in which differences in behaviour could affect susceptibility may be different to non-infectious conditions; differences in behaviour which might affect the incidence of cuts and abrasions on the heels, the amount of contact with infected animals, or the amount of contact with reservoirs of infection could all be significant.

Only one study to date has specifically examined dairy cow behaviour and susceptibility to DD. Palmer and O’Connell [116] monitored Holstein-Friesian dairy cows in a cubicle house with solid concrete floors, comparing the behaviour of those that subsequently developed DD with those that did not. They found two sets of behaviours that were performed more by animals that went on to develop DD; standing and walking in the passageway and crosswalks (short passages between the passageway and feed face), and standing half in the cubicle. The authors suggested that more time spent in the passageways could increase the duration of contact between the heels and slurry, possibly increase interactions with infectious animals and may also increase the amount of conflicts with other animals, which could lead to damage to the heel skin. An increased amount of time spent standing half in a cubicle or ‘perching’ has been associated with the development of lameness and claw horn lesions in previous research [114,115,117]. It has been suggested that standing half in a cubicle could reduce heel height, predisposing animals to infection [114] and it is also likely to increase the contact time between the rear hooves and both slurry and concrete [117]. In addition to behaviour within the housing area of dairy cows, the amount of time that they spend in contact with slurry when standing waiting to be milked could also be of importance. The duration of time spent waiting to be milked can vary greatly between animals within a herd, depending on their position in the milking order.

### 5.7. Coping Strategies, Personality and Social Status

Individual susceptibility to disease, including DD, might also be affected by how an animal deals with social stress induced by housing and management practices [118]. How animals are affected by a physiological stress response, including effects on the immune system, can vary depending on their personality, coping strategy and social status (reviewed by [118]). It appears that individuals with certain personality types are at higher risk of developing particular pathologies due to the nature of their physiological stress responses [118]. For example, Cohen *et al.* [119] found that people who were more sociable were less susceptible to infection by the common cold virus. The relationship between personality, immune system function and disease susceptibility is complex, however, due to the many processes acting concurrently on an individual [120].

The majority of the literature examining relationships between coping style and physiological stress responses refers to research carried out in laboratory rodents, with a small amount also carried out in pigs [121]. Two differing coping styles (or strategies) are described; a more proactive style associated with a high sympathetic reactivity to stressors, and a more passive (or reactive) style which is typically associated with higher hypothalamic–pituitary–adrenal axis reactivity [121]. Although there is not a great deal of evidence for the relationship between coping styles and susceptibility to disease, the two coping styles have been associated with differences in immune response in pigs [122] and with different immune responses to, followed by differing susceptibility to, an experimentally induced autoimmune disease in rats [123].

The relationship between social status and immunity appears to be linked to exposure to social stressors; for example individuals in a stable group who experience more defeats during social conflicts (and are therefore considered to have low status) have a poorer immune response and are more susceptible to infectious disease [118]. Social status is a less reliable method of categorising animals than personality or coping strategy, however, as social status can vary between contexts [118]. Although research into the relationships between personality types, coping styles, social status and susceptibility to disease has not yet provided clear answers, it is an area that could provide interesting insights into individual differences in susceptibility to different diseases including DD.

## 6. Conclusions

Overall, considerable progress has been made in DD research over recent decades, particularly in key areas such as the identification of causative agents and reservoirs of infection. There is, however, still no ‘cure’ or effective vaccine for DD, so the problems of reduced animal welfare and reduced farm profits remain. A number of factors have been identified which affect DD risk at a farm, animal and individual level. The research into farm level risk factors has identified higher risk housing and management practices, and the research into animal level factors has identified groups of animals which are at higher risk of infection. This information could help farmers to alter their management (where possible) in order to reduce the prevalence of DD on their farms. The number of interacting risk factors at different levels and the infectious nature of DD, however, mean that the factors which are most important could vary between farms and over time.

In terms of factors affecting individual susceptibility, there is some evidence that differences in hoof conformation and behaviour might affect susceptibility to DD. The role that differences in properties of the skin and density of hair follicles could play in susceptibility to DD infection may become clearer as more is learned about the mechanisms of DD transmission between animals and the establishment of DD infections. Investigating the immune response to DD is a complex task due to the variety of bacteria that have been isolated from lesions and the finding that at least some of the DD associated treponemes can modify the host immune response [105,112]. Individual differences in the immune response have been found to affect susceptibility to a number of other infectious diseases in dairy cows [76], and a number of the SNPs identified as differing between DD susceptible and non-susceptible animals by Scholey *et al.* [88] were in regions related to inflammation, therefore it seems likely that immune differences in susceptibility to DD may be detected in the future.

Further work is required to identify the genetic basis of individual variation in susceptibility, to clarify the level of heritability of DD susceptibility and to determine how this is correlated with production and health traits currently used in breeding programmes. Research into the effects of social stressors on animals with different personality types, coping strategies and positions in the social hierarchy could help to inform management strategies which might reduce susceptibility to infectious diseases in general [118]. In addition to this, research to confirm the role of behavioural differences in susceptibility to DD could lead to work examining whether changes in the design of housing might reduce DD prevalence by not only aiming to provide a drier and more hygienic environment for the feet, but also by reducing the proportion of animals standing in passageways and half in cubicles (behaviours found to be associated with DD infection by Palmer and O’Connell [116]). Research into phenotypic and genetic differences between susceptible and non-susceptible individuals, combined with the results of continuing research into the etiology and transmission of the disease, could help in the development of breeding and herd management strategies aimed at reducing the prevalence of DD.
